# Fistule artério-veineuse iatrogène carotido-jugulaire: une complication rare du cathétérisme de la veine jugulaire (à propos d’un cas)

**DOI:** 10.11604/pamj.2020.37.379.26146

**Published:** 2020-12-24

**Authors:** Othman Zahdi, Noured-dine Lahlou, Hajar El Bhali, Mohamed Hormat-Allah, Samir El Khloufi, Yasser Sefiani, Abbes El Mesnaoui, Brahim Lekehal

**Affiliations:** 1 Université Mohammed V de Rabat, Rabat, Maroc,; 2 Service de Chirurgie Vasculaire, Centre Hospitalo-Universitaire Ibn Sina, 10104 Souissi, Rabat, Maroc

**Keywords:** Fistule artério veineuse, artère carotide, veine jugulaire, cathéter, *case report*, Arterio-venous fistula, carotid artery, jugular vein, catheter, case report

## Abstract

Le cathétérisme de la veine jugulaire interne est pratiqué largement, aussi bien par les néphrologues que par les chirurgiens, il est devenu une procédure de routine, mais qui peut être associée à de graves complications. La fistule artério-veineuse carotido-jugulaire (FCJ) est une complication rare mais potentiellement fatale. Très peu de cas sont rapportés dans la littérature. Nous présentons un cas unique d´un patient avec une FCJ iatrogène, suite à une pose de cathéter d'hémodialyse trois semaines auparavant. Le diagnostic est suspecté cliniquement par la découverte de thrill latéro-cervical, et confirmé à l´écho-Doppler et à l´angiographie. Le patient a eu une cure chirurgicale ouverte avec succès. L'expérience de l´opérateur et l´accompagnement des jeunes internes, en plus de l'utilisation du guidage échographique peut réduire considérablement la survenue de complications lors du cathétérisme veineux jugulaire.

## Abstract (en)

Catheterization of the internal jugular vein is widely performed by both nephrologists and surgeons. It has become a routine procedure, but can be associated with serious complications. Carotid-jugular arterio-venous fistula (CJAVF) is a rare but potentially fatal complication. Very few cases have been reported in the literature. This study involved a patient with iatrogenic CJAVF following hemodialysis catheter placement performed three weeks earlier. The diagnosis was clinically suspected based on the discovery of laterocervical thrill and confirmed by echo-Doppler and angiography. The patient underwent successful open surgery. Operator experience and trainees support, in addition to the use of ultrasound guidance, can significantly reduce the occurrence of complications during jugular venous catheterization.

## Introduction

Le nombre de patients présentant une insuffisance rénale est en constante augmentation ces dernières années, ceci est accompagné d´une utilisation de plus en plus fréquente, de cathéters veineux centraux de comme accès vasculaire temporaire ou parfois permanent [1]. La veine jugulaire interne est le site le plus souvent utilisé. La survenue de complications est non négligeable malgré l´amélioration des techniques de pose (guidage échographique, scopie). Nous rapportons un cas rare de fistule carotido-jugulaire (FCJ) iatrogène suite à la mise en place d'un cathéter d'hémodialyse à double lumière, traitée chirurgicalement avec succès.

## Patient et observation

Un patient de 57 ans, avec une insuffisance rénale chronique sur lithiase rénale, commence l´hémodialyse par un cathéter jugulaire, dans l´attente de la création et la maturation d´une fistule artério-veineuse native. Un cathéter tunnelisé jugulaire droit est posé sans incident, avec une ponction veineuse aidée par un marquage échographique préalable. Le patient présente trois semaines plus tard une gêne cervicale, l´examen physique retrouve un thrill latéro-cervical droit, sans déficit neurologique ou signes d´insuffisance cardiaque. Le diagnostic de FCJ est suspecté.

**Figure 1:**
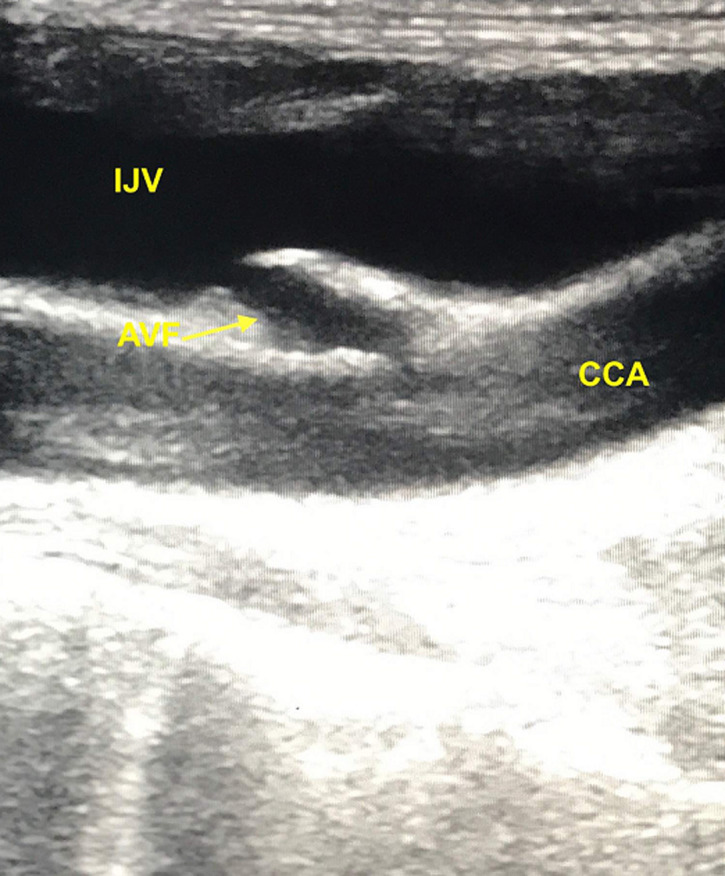
image échographique montrant la communication directe entre l´artère carotide commune et la veine jugulaire interne

**Figure 2:**
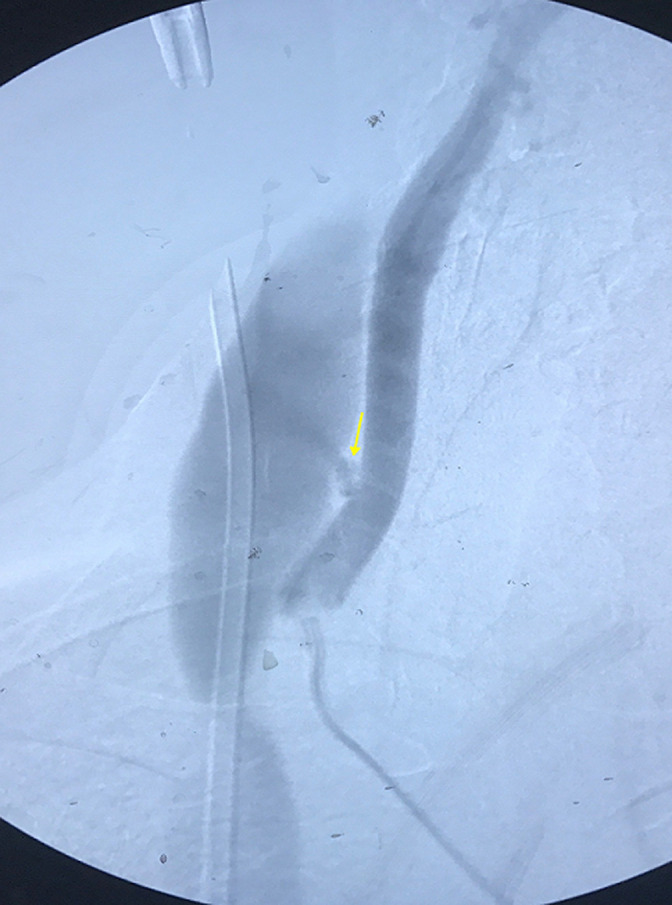
angiographie montrant la fistule artério-veineuse et l'opacification rapide de la veine jugulaire interne

Un écho-Doppler couleur montre la présence d´une fistule artério veineuse faisant communiquer la veine jugulaire interne et l´artère carotide commune (Figure 1), avec enregistrement d´un flux artérialisé dans la veine. Le diagnostic de FCJ est confirmé par une angiographie trans-radiale, en effet, une opacification précoce et rapide de la veine jugulaire interne droite, ainsi que la communication artério veineuse est visualisée (Figure 2).

**Figure 3:**
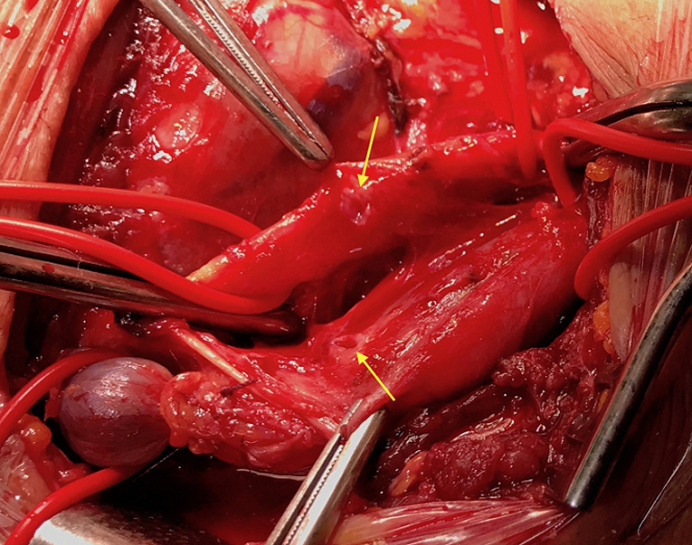
vue peropératoire montrant les orifices qui constituaient la fistule sur les parois de l'artère et de la veine

Une cure chirurgicale ouverte de la FCJ est réalisée, ceci a consisté en une excision de la fistule, après un contrôle proximal et distal de l´artère carotide commune et de la veine jugulaire interne de part et d´autre de la communication. Un orifice de 4 mm est découvert sur la paroi des deux vaisseaux (Figure 3), qui est réparé par des sutures latérales en polypropylene, après un clampage carotidien d´environ cinq minutes. Le suivi post-opératoire ne retrouve pas de complications neurologiques ou cardiaques, le patient est revu à trois puis six mois avec un écho-Doppler cervical de contrôle, qui est sans particularité.

## Discussion

Les traumatismes iatrogènes suite à la mise en place d'un cathéter jugulaire interne, sont des incidents graves et potentiellement fatals. Ils comprennent la perforation veineuse, le pneumothorax, la tamponnade cardiaque, le syndrome de Horner et les lésions thyroïdiennes, mais aussi les lésions de l'artère carotide, qui peuvent entraîner d'autres complications graves, telles que l'hémothorax ou la formation de faux anévrisme ou d´une FCJ [1]. Bien que la ponction accidentelle de l'artère carotide soit la complication la plus courante, les FCJ sont rarement rapportées [2]. En effet, dans une revue de la littérature, Caldarelli *et al*. n'a rapporté que 14 cas de FCJ iatrogène [3]. Les facteurs prédisposant à la formation de la fistule sont les lésions pariétales artérielles, telles que les anévrismes et les calcifications, associées à diverses maladies systémiques, telles que les maladies du tissu conjonctif [4].

L'artère carotide externe est moins fréquemment touchée, par rapport à l'artère carotide commune et à l'artère carotide interne, du fait de sa plus petite taille et de sa position plus interne au niveau de la bifurcation carotidienne chez la plupart des patients [5]. La réparation précoce, est considérée comme bénéfique par de nombreux auteurs [6]. En effet si elle n'est pas traitée, la fistule peut entraîner une insuffisance cardiaque congestive, des arythmies cardiaques ou des événements thromboemboliques cérébraux [7]. Le choix thérapeutique varie entre la réparation chirurgicale ouverte ou bien endovasculaire, cependant la supériorité d´une technique sur l'autre reste controversée [8]. Le traitement endovasculaire consiste en l´exclusion de la FCJ via l´insertion d´un stent couvert au niveau de l'artère carotide, ou bien par embolisation par micro-coils si la fistule est de petit diamètre. Il s'agit d'une option thérapeutique moins invasive, mais elle comporte des risques thromboemboliques, surtout en cas de lésions athéromateuses associées au niveau de l´arche aortique.

La réparation chirurgicale ouverte était le traitement de première intention avant l´avènement et le développement des techniques endovasculaires, nous pensons qu´elle garde encore sa place comme stratégie thérapeutique sûre et efficace, pour cette entité très rare, compte tenu de l´accessibilité des vaisseaux cervicaux et du taux plus faible de complications thromboemboliques. Il n'y a pas de séries de cas ou d'études de suivi à long terme comparant les deux techniques pour cette entité. Cependant, la chirurgie semble la meilleure option en cas de fistules complexes ou de grande taille, ou lorsqu'elles sont associées à un faux anévrisme ou à d'autres lésions vasculaires, ou encore en cas d´échec de procédures endovasculaires [9]. L'utilisation du guidage échographique est indispensable pour réduire le risque de complications iatrogènes [10]. Le bon positionnement de la tête est aussi un point technique important : il est nécessaire de limiter la rotation externe à la ponction, pour éviter une superposition de la veine jugulaire avec l'artère carotide [11]. Enfin, il est recommandé de ne pas multiplier les tentatives de ponction par le même opérateur, le risque de survenue de complication est six fois plus élevé au-delà de la troisième tentative [12].

## Conclusion

Le cathétérisme de la veine jugulaire interne est une pratique courante, pour les accès vasculaire d´hémodialyse. La FCJ est une complication certes rare, mais grave. Le traitement doit être rapide; il peut être chirurgical ou endovasculaire. L´expérience de l'opérateur et l´accompagnement durant la formation des jeunes médecins, ainsi que le guidage échographique systématique peuvent réduire la survenue de complications.
